# Pharmacokinetics of Oral Cholecalciferol in Healthy Subjects with Vitamin D Deficiency: A Randomized Open-Label Study

**DOI:** 10.3390/nu12061553

**Published:** 2020-05-27

**Authors:** Angelo Fassio, Giovanni Adami, Maurizio Rossini, Alessandro Giollo, Cristian Caimmi, Riccardo Bixio, Ombretta Viapiana, Stefano Milleri, Matteo Gatti, Davide Gatti

**Affiliations:** 1 Rheumatology Unit, University of Verona, 37131 Verona, Italy; adami.g@yahoo.com (G.A.); maurizio.rossini@univr.it (M.R.); alessandrogiollo@gmail.com (A.G.); cristian.caimmi@gmail.com (C.C.); dott.riccardobixio@gmail.com (R.B.); ombretta.viapiana@univr.it (O.V.); matgat93@gmail.com (M.G.); davide.gatti@univr.it (D.G.); 2 Centro Ricerche Cliniche di Verona SRL, 37131 Verona, Italy; stefano.milleri@crc.vr.it

**Keywords:** vitamin D, cholecalciferol, osteoporosis, osteomalacia, supplementation

## Abstract

Background: The aim of this study was to investigate the pharmacokinetic (PK) and safety profile of high-dose vitamin D supplementation, comparing different schedules (daily, weekly, or bi-weekly) in an otherwise healthy vitamin D-deficient population. Methods: Single-center, open-label study on healthy subjects deficient in vitamin D (25 (OH)D < 20 ng/mL), randomized to receive cholecalciferol (DIBASE^®^, Abiogen Pharma, Italy) using three different schedules: Group A: 10,000 IU/day for eight weeks followed by 1000 IU/day for four weeks; Group B: 50,000 IU/week for 12 weeks, Group C: 100,000 IU/every other week for 12 weeks. Total cumulative doses were: 588,000 IU, 600,000 IU, 600,000 IU. The treatment regimens corresponded to the highest doses allowed for cholecalciferol for the correction of vitamin D deficiency in adults in Italy. Results: mean 25 (OH)D plasma levels significantly increased from baseline 13.5 ± 3.7 ng/mL to peak values of 81.0 ± 15.0 ng/mL in Group A, 63.6 ± 7.9 ng/mL in Group B and 59.4 ± 12 ng/mL in Group C. On day 28, all subjects showed 25 (OH)D levels ≥20 ng/mL and 93.1% had 25 (OH)D levels ≥30 ng/mL. On day 56 and 84, all subjects had 25 (OH)D levels ≥30 ng/mL. No serious adverse events occurred during the study. Conclusions: normalization of 25 (OH)D serum levels was quickly attained with all the studied regimens. A more refracted schedule provided a higher systemic 25 (OH)D exposure.

## 1. Introduction

The term “vitamin” defines a trace dietary constituent required for the normal function of specific physiological processes [1]. Vitamin D is essential for the correct regulation of calcium-phosphorus homeostasis and maintenance of the musculoskeletal system [2].

Two types of vitamin D molecules exist: vitamin D3 (cholecalciferol), which is animal-derived; and the plant-derived vitamin D2 (ergocalciferol). Normally, the body is unable to metabolically synthesize vitamins but an important peculiarity of vitamin D is its photochemical production [1]. Vitamin D can be endogenously synthesized from sterol 7-dehydrocholesterol in the skin following exposure to ultraviolet B radiation (from 80% to 90% of body requirement). Alternatively, vitamin D, in the form of either vitamin D3 or D2, can be acquired from the diet or dietary supplements. This source of vitamin D is essential when sun exposure or the response of skin to ultraviolet radiation is insufficient, such as in elderly people. Indeed, the skin content of 7-dehydrocholesterol decreases with age together with the efficiency of the human skin to synthesize vitamin D3 [3].

Vitamin D, either as D3 or D2, requires a two-step activation process to become biologically active. Vitamin D3 is transported in the bloodstream bound to a specific plasma protein: vitamin D-binding protein (VDBP). It is taken up within hours following synthesis or dietary uptake to be activated mainly by the liver to 25 (OH)D and, then, mainly by the kidney to 1, 25 (OH) D (calcitriol) [4].

At present, serum 25 (OH)D levels are considered the best measure of vitamin D status [5].

Several epidemiological studies have shown that vitamin D deficiency is highly prevalent worldwide, particularly in the elderly [6,7]. Many observational studies have linked low vitamin D serum levels to major human diseases [8,9], however, interventional studies for extra-skeletal health are still inconclusive [9] and are often affected by methodological issues [10].

In the setting of bone disease, it is widely recognized that low and very low vitamin D levels (i.e., below 20 and 10 ng/mL respectively) have deleterious effects on skeletal health [2], leading to a number of science organizations or expert groups advocating for serum concentrations of 25 (OH)D >30 ng/mL [2].

In addition, there is an even greater lack of consensus about the recommended regimen for vitamin D supplementation (doses, administration schedule, treatment duration, etc.) [2].

This heterogeneity can be explained, at least in part, by the scarcity of comparative pharmacokinetics (PK) data for the different dosing schedules [11,12,13]. Furthermore, there is growing evidence suggesting that the treatment schedule itself (i.e., bolus vs. frequent administration) may impact differently on the effectiveness of the treatment [14,15] and also on clinical outcome, with recent studies and few meta-analyses showing more promising results with frequent administration schedules on skeletal and extra-skeletal outcomes [9,10,16,17,18].

The aim of this open-label randomized clinical trial was to compare the PK and safety profile of high-dose vitamin D supplementation, administered with three different treatment schedules (daily, weekly, or bi-weekly) in an otherwise healthy vitamin D deficient population.

## 2. Materials and Methods

This was a single-center, open-label, randomized, parallel-group study undertaken in male and female healthy subjects to compare the PK profiles of an approximately equal amount of cholecalciferol (DIBASE^®^, Abiogen Pharma, Italy) administered using three different schedules: Group A: repeated once daily [10,000 International Units (IU)/day] for 8 weeks followed by 1000 IU daily for 4 weeks; Group B: weekly (50,000 IU/week for 12 weeks) and Group C): bi-weekly doses (100,000 IU every other week for 12 weeks).

The regimens adopted in this trial correspond to the highest doses allowed for cholecalciferol (DIBASE^®^) in Italy, according to its Summary of Product Characteristics (SmPC), for the correction of vitamin D deficiency in adults [19]. The only deviation from SmPC dosing schedules occurred in Group A, where the 1000 IU daily dose for 4 weeks is not indicated in the SmPC; however, these were considered necessary to guarantee almost the same total vitamin D intake across all groups.

The date of the first enrollment visit was 17 October 2017, and the date of the last enrolment visit was 14 February 2018. The date of the last completed visit was 5 June 2018.

This study was approved by the institutional research committee (protocol identification: DIBA/11, EudraCT Number: 2017-000194) and was conducted in accordance with the 1964 Helsinki declaration and its later amendments or comparable ethical standards. Written informed consent was obtained from all individual participants included in the study.

The primary objective of this study was to compare the pharmacokinetic profiles of cholecalciferol (DIBASE^®^) administered as repeated once daily, weekly and bi-weekly doses in healthy male and female volunteers.

Pharmacokinetic parameters determined from the serum 25 (OH)D concentrations using non-compartmental procedures are listed below:

1. Cmax: Maximum observed serum concentration
2. AUC0-84: Area under the serum concentration × time curve from day 0 to day 84
3. AUC0-112: Area under the serum concentration × time curve from day 0 to day 112
4. AUC84-112: Area under the serum concentration × time curve from day 84 to day 112

Secondary objectives of this study were:

(1) To assess the rate of subjects showing 25 (OH)D levels ≥20 ng/mL and ≥30 ng/mL at a steady state and the safety and tolerability of cholecalciferol.

(2) To assess the changes to phosphate-calcium values after administration of DIBASE^®^ as repeated once daily, weekly and bi-weekly doses in healthy volunteers.

Inclusion criteria were: otherwise healthy Caucasian female or male participants aged 18–60 years, BMI between 18.5 kg/m^2^ and 28 kg/m^2^; screening 25 (OH)D <20 ng/mL; negative urine pregnancy test. Exclusion criteria were: excessive consumption of tea, cocoa, coffee and/or beverages containing caffeine (>5 cups/day) or wine (>0.5 L/day) or spirits (>50 mL/day) on a regular basis; substantial changes in eating habits in the previous 4 weeks; use of any medicinal product (including glucocorticoids, anticonvulsants, antibacterial drugs, over-the-counter medication, vitamins, and natural products) in the previous 14 days; blood donation of 250 mL or more within the past 3 months; history of clinically significant gastrointestinal, renal (including renal stone formation), hepatic, pulmonary, endocrine, oncologic, or cardiovascular disease; or history of epilepsy, asthma, diabetes mellitus, psychosis or severe head injury; vitamin D therapy or food supplements applied within two months; metabolic disorders of calcium or bones (including secondary hyperparathyroidism), history of angina pectoris, artificial ultraviolet B-rays (UVB) exposure (solarium) in the previous 2 weeks.

After screening, eligible individuals were randomized in a 1:1:1 ratio, in which subjects had an equal probability of being assigned to one of the three treatments. Supplementation was taken with the main meal of the day, regardless of the treatment group and at the same time of day. In order to assess compliance, subjects were provided with a diary to record the intake of the study drug (daily, weekly, or bi-weekly schedule). Subjects had to return the used/unused supplementation and the diary on the next treatment-delivering occasion.

As already specified, treatment with different schedules was administered up to day 84 (week 12) and then discontinued, while follow-up was maintained up to day 112. In all three groups, blood samples for PK analysis were collected pre-dose on each of days 1, 7, 14, 21, 28, 35, 42, 49, 56, 63, 77, 84 during the treatment period and on each of days 85, 87, 89, 96, 103, and 112 of the follow-up period (treatment-free).

Blood samples for calcium, albumin, and phosphate were collected pre-dose on day 1, 28, 56, 84 and 112.

Blood samples collected at each time point were processed (by centrifugation for 15 min at 4 °C) to obtain serum samples. These samples were stored at −70 °C until the end of the study when all of them were analyzed by the LIAISON^®^ 25 OH Vitamin D assay (DiaSorin, Italy), a direct competitive chemiluminescent immunoassay for the quantitative determination of total 25 (OH)D in serum, that was used for the PK assessment. The intra-assay variation coefficient was 8% and the inter-assay variation coefficient was 12%.

## 3. Statistical Analysis

Statistical analysis was performed by Advice Pharma Group S.r.l., Milano, Italy, using SPSS software, Version 22 (SPSS, Inc., Chicago, IL, USA).

Analysis of variance (ANOVA) with post-hoc analysis (Bonferroni) and a two-sided Student’s *t*-test was used to estimate the absolute differences between groups. Two-sided *p*-values of 0.05 or lower were considered statistically significant. Data are presented as mean ± SD.

To compare the pharmacokinetic profiles of DIBASE^®^ administered as repeated once daily, weekly and bi-weekly doses in healthy male and female volunteers, the ratios with associated 90% confidence intervals (CI) for Cmax, AUC0-84, AUC0-112, and AUC84-112 obtained using the residual variance from the analysis of variance on log-transformed variables, were calculated using 1-way ANOVA for each of the dose groups, The sample size of 25 subjects per arm was primarily based on clinical judgment and practical considerations and not on formal statistical reasoning. However, the selected sample size of 25 subjects per group would allow detection of a change in trough 25(OH)D concentration from baseline of 16 ng/mL with a statistical power of 80% and a Type I error of 0.05. In addition, with 25 subjects per group, there was an 80% probability of detecting an adverse event (AE) with an underlying incidence rate of 0.07.

## 4. Results

### 4.1. Baseline Characteristics

**Figure 1:**
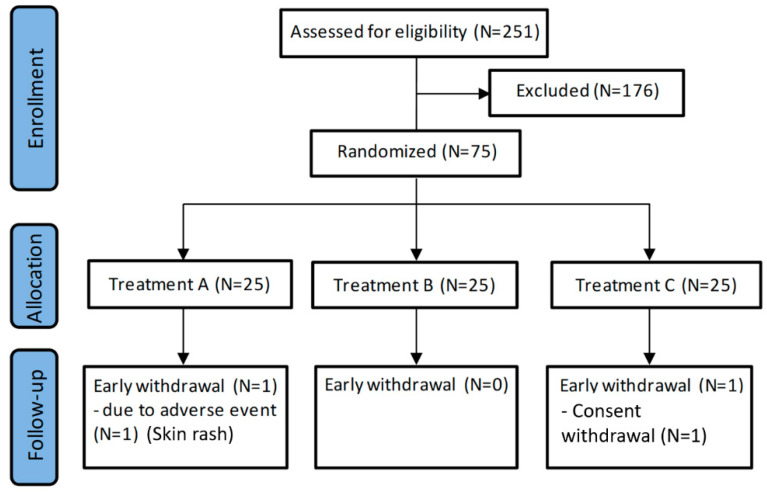
Flowchart of the study.

In total, 251 healthy volunteers were screened for eligibility. Of these, 75 subjects were randomized to treatment (25 in each treatment arm) (Figure 1).

Of the 75 randomized subjects, 73 volunteers completed the study and 2 prematurely discontinued. One subject in Group A discontinued due to an AE (reported as skin rash with mild severity and not related to the treatment), and 1 subject in Group C discontinued due to withdrawal of consent.

Demographic and biochemical characteristics at baseline are reported in Table 1. No significant difference between treatment groups was observed at baseline for any of the reported parameters. Compliance to treatment was 100% for each group at each evaluation.

### 4.2. Pharmacokinetic Parameters

**Figure 2:**
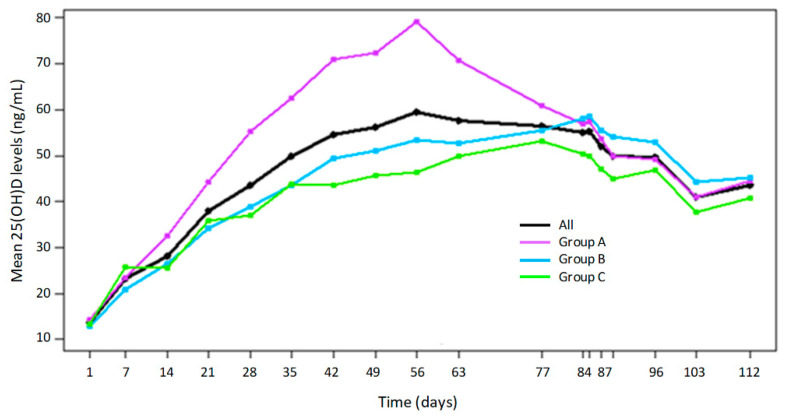
Serum 25 (OH)D levels in healthy participants treated with the three different dosing regimens of cholecalciferol over the study period. Group A; 10,000 International Units (IU) daily, Group B; 50,000 IU weekly and Group C; 100,000 IU bi-weekly doses of cholecalciferol.

The PK parameter analysis for 25 (OH)D following repeated oral doses of cholecalciferol is reported in Table 2, Figure 2, and supplementary material Table S1.

Twenty-five (OH)D plasma levels increased from an average baseline value of 13.5 ± 3.7 ng/mL to peak values of 81.0 ± 15.0 ng/mL in Group A, 63.6 ± 7.9 ng/mL in Group B and 59.4 ± 12 ng/mL in Group C. Maximal plasma levels were achieved on Day 56 in Group A and on Days 85 and 81 in Groups B and C, respectively. Following the last dose, plasma levels then decreased to a similar extent across the three groups.

Systemic exposure (AUC0-84 and AUC0-112) to 25 (OH)D was higher in Group A compared to Groups B and C), even though the cumulative dose of cholecalciferol was similar among groups (588,000 IU in Group A vs. 600,000 in Groups B and C). Similarly, Cmax values for Group A were higher than for Groups B and C. Following the last dose until the last day of sampling, systemic exposure (AUC84-112) was similar across groups.

After correcting geometric means of AUCs for the cumulative dose reached up to the respective time point then compared among the different groups, we observed the following data. Time interval day 0 to 56, no significant difference was found among the groups: A vs. B 0.97 (95% CI: 0.91–1.04), A vs. C 1.01 (95% CI: 0.94–1.09), B vs. C 1.05 (95% CI: 0.97–1.12). Time interval day 0 to 84 (treatment period), Group A showed a significantly higher value than the other two groups, and Group B showed a slightly higher value than C: A vs. B 1.32 (95% CI: 1.22–1.42), A vs. C 1.41 (95% CI: 1.31–1.51), B vs. C 1.07 (95% CI: 0.99–1.15). Time interval day 0 to 112 (treatment plus follow-up period) A vs. B 1.21 (95% CI: 1.13–1.32), A vs. C 1.34 (95% CI: 1.24–1.44), B vs. C 1.10 (95% CI: 1.02–1.19). Time interval day 0 to 112, Group A showed a significantly higher value than the other two groups and Group B showed a significantly higher value than C: A vs. B: 1.21 (95% CI: 1.13–1.32), A vs. C: 1.34 (95% CI: 1.24–1.44), B vs. C: 1.10 (95% CI: 1.02–1.19).

### 4.3. Achievement of 25 (OH)D ≥20 ng/mL and ≥30 ng/mL Levels

**Figure 3:**
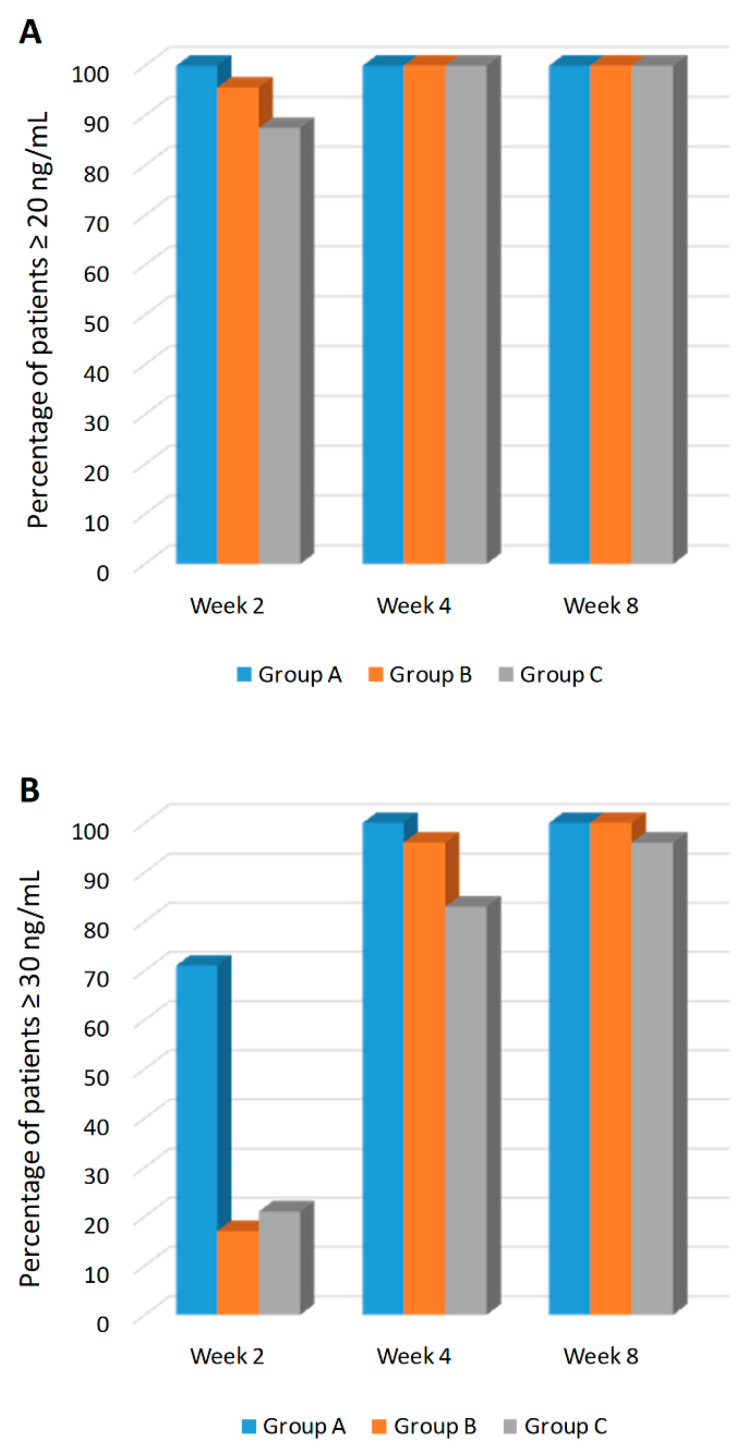
Percentage of patients achieving the 20 ng/mL (**A**) and 30 ng/mL threshold (**B**) at weeks 2, 4, and 8. Group A; 10,000 IU daily, Group B; 50,000 IU weekly and Group C; 100,000 IU bi-weekly doses of cholecalciferol.

**Figure 4:**
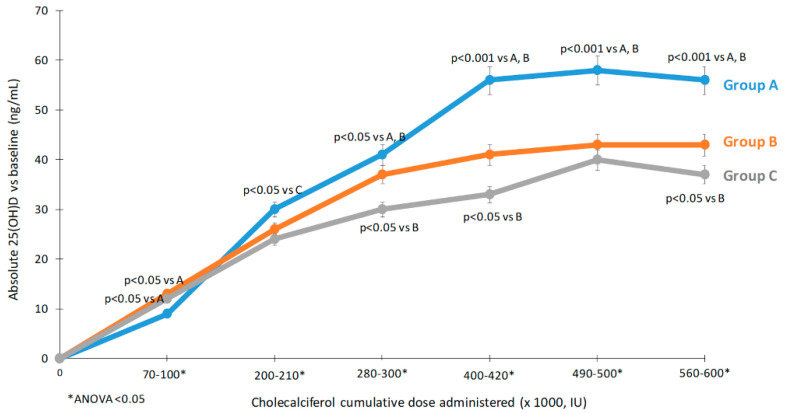
Relationship between the 25 (OH)D increase with respect to the cumulative dose reached among the three different groups. Group A 10,000 IU daily, Group B 50,000 IU weekly, Group C 100,000 IU biweekly. * ANOVA *p* < 0.05. Group A; 10,000 IU daily, Group B; 50,000 IU weekly and Group C; 100,000 IU bi-weekly doses of cholecalciferol. Data presented as mean ± SD.

The proportion of individuals with 25 (OH)D levels ≥20 ng/mL and ≥30 ng/mL at each observation point is reported in Supplementary Material Table S2 and Figure 3. As early as day 14, 100% of subjects in Group A reached 25 (OH)D levels ≥20 ng/mL (Figure 3A). On day 28, all subjects showed 25 (OH)D levels ≥20 ng/mL, and this was maintained through day 112. On day 28, 66 subjects (93.1%) evaluated had 25 (OH)D levels ≥30 ng/mL (24 subjects in Group A, 22 subjects in Group B, and 20 subjects in Group C) (Figure 3B). On day 56, and day 84, all subjects had 25 (OH)D levels ≥30 ng/mL. On day 112, 66 subjects (98.5%) showed levels of 25 (OH)D ≥30 ng/mL (24 subjects in Group A, 22 in Group B, and 21 in Group C). The relationship between the 25 (OH)D increase with respect to the cumulative dose reached among the three different groups are presented in Figure 4.

### 4.4. Changes in Calcium, Phosphate and Albumin

Pharmacodynamic parameters over time for calcium, phosphate, and albumin are presented in Supplementary Material Table S3.

Statistically significant changes in albumin were found with respect to baseline only for Group B at day 56 (+1.9%, *p* = 0.0046).

A statistically significant, but clinically irrelevant, increase in serum total calcium was found with respect to baseline for Group B at day 56 (+1.9%, *p* < 0.001), day 84 (+1.7%, *p* = 0.021) and day 112 (+1.5%, *p* = 0.018). When all subjects from the three groups were pooled together, the change was significant at day 56 (+1.4%, *p* = 0.002), day 84 (+0.8%, *p* = 0.036) and day 112 (+1.1%, *p* = 0.008). A consensual significant change in albumin (increase) matches with the increase serum calcium only in Group B at day 56.

A statistically significant increase in phosphate was found with respect to baseline only at day 28 for Group C (+6.4%, *p* = 0.0047). When all subjects were pooled together, the change was significant at day 28 (+4.5%, *p* = 0.009), day 56 (+3.8%, *p* = 0.032) and day 84 (+4.5%, *p* = 0.008%).

### 4.5. Safety

During the study, 49 subjects (65.3%) had an AE (for a total of 118 adverse events): 18 subjects (72.0%) in Group A, 19 subjects (76.0%) in Group B, and 12 subjects (48.0%) in Group C.

Most of the AEs observed were mild in intensity reported in 38 subjects (50.7%), 25 subjects (33.3%) were reported with moderate adverse events (AEs), and none of the AEs were severe.

No deaths, serious adverse events (SAEs), or severe AEs occurred during the study.

Only one subject (1.3%) in Group A reported an AE considered to be study drug-related (constipation of mild intensity).

No cases of hypercalcemia (i.e., total serum calcium >2.63 mmol/L) were recorded.

The summary of AEs occurring during the study is provided in Supplementary Material Table S4.

## 5. Discussion

This is the first randomized study comparing the pharmacokinetics of cholecalciferol supplementation with three different regimens corresponding to the highest doses allowed for cholecalciferol, according to its Summary of Product Characteristics (SmPC), for the correction of vitamin D deficiency in adults [19].

In this study, we showed that, after the first month of treatment, all subjects successfully exceeded the threshold of 20 ng/mL. In particular, in the group receiving daily administration of 10,000 IU/day, the threshold was achieved in all subjects within two weeks.

Regardless of the treatment regimen, the vast majority of subjects (93%) had 25 (OH)D serum levels ≥30 ng/mL after the first month and in two months the percentage increased to 100%. Overall, these findings demonstrate that all three regimens grant a quick repletion of serum 25 (OH)D levels in deficient subjects and suggest that longer loading phases are probably unnecessary.

Our study confirmed that the use of higher doses of vitamin D for 8–12 weeks is not associated with the appearance 25 (OH)D serum levels potentially at risk for hypercalcemia (>100 ng/mL) [20,21,22,23].

Another important result that emerged from our study was that daily administration was associated with a higher systemic exposure to 25 (OH)D (greater AUC, also corrected for the cumulative dose).

Clinical practice aside, our results concerning the pharmacokinetic profiles of cholecalciferol supplementation contribute to our understanding of vitamin D metabolism. Vitamin D supplementation is usually expressed as a daily dose, but in the real-life scenario similar cumulative doses are often administered with weekly, biweekly, or monthly boluses for compliance purposes. The rational of bolus-based regimens has usually been based on the assumption that, after a bolus, the lipophilic cholecalciferol would be quickly stored in the adipose tissue, explaining its long-term availability to the organism [24]. The results of this study show that (at equal cumulative doses) the daily administration is more efficient (in terms of 25 (OH)D exposure) than the weekly one, which, in turn, is slightly more efficient than the bi-weekly supplementation schedule, presumably due to a different involvement of the enzymatic compartment (i.e., higher efficiency or lower saturation of 25-hydoxylase enzyme or lesser induction of the vitamin D-catabolizing enzyme 24-hydroxylase) [14]. This is consistent with a previous observation that showed a greater production of 24,25 (OH)2D with the bolus-based regimen than daily supplementation [14].

Previously, Wylon et al. [11] studied the effects of daily oral cholecalciferol administration of 2000 to 8000 IU for 12 weeks (total cumulative dose 392,000 IU distributed in 84 days, mean daily dose 4666 IU). In this cohort there were also patients with normal 25 (OH)D serum levels at baseline. This detail should be emphasized, because these findings demonstrate that the increase in 25 (OH)D serum levels during cholecalciferol supplementation follow a biphasic curve: a rapid increase usually seen in deficient states while a slower response when concentrations are higher [25]. In that study, peak serum levels of 25 (OH)D of the group treated with the oral regimen were similar to the peak serum levels of Groups B and C in our study (64 ng/mL vs. 63.17 and 58.27 ng/mL, respectively), although in our study the total cumulative dose was greater (588,000 IU vs. 600,000 IU). Altogether, this findings and ours suggest a higher efficiency of the frequent administration when compared to bolus-based regimens in increasing 25 (OH)D levels and finds further support also from our post-hoc analysis in which we compared 25 (OH)D levels reached in the three different treatment schedules for given cumulative doses (Figure 4) and comparison of AUCs corrected for cumulative doses.

It is nonetheless interesting to compare our data to those derived from previous studies [25,26]. Overall, the evidence seems to show a tendency to plateau once levels of serum 25 (OH)D of 30–40 ng/mL are exceeded, probably due to the upregulation of enzymes involved in the negative control of 25 (OH)D [27]. In this manner, once again, the efficiency of cholecalciferol supplementation in replete patients is being physiologically reduced by the organism, arguably to avoid intoxication. This mechanism is probably a safeguard that prevents people living in sun-rich conditions from exceeding serum 25 (OH)D levels of 40–60 ng/mL [28]. In order to overcome these thresholds, it seems that higher and more frequent doses might be needed (i.e., Group A up to day 84), and with a quick decrease after the switch to a relatively low dose: from daily 10,000 IU to daily 1000 IU, respectively.

A similar biphasic response has also been observed in a study by Rahme et al. [13], in which the high dose cholecalciferol group (daily equivalent of 3750 IU for 12 months, but the actual schedule was based on a weekly bolus-based one) showed a substantial increase in the first six months and then plateaued at around 36 ng/mL. Similarly, De Niet et al. [26] also compared a daily and a bolus-based regimen (2000 IU daily for 75 days and 50,000 IU every 25 days) in deficient subjects and observed a peak 25 (OH)D concentration of roughly 28 ng/mL, once again with a steeper curve in the first weeks (with a statistically significant difference in favor of the daily regimen in the first two weeks). On the other hand, in that study, while succeeding in attaining 25 (OH)D levels >20 ng/mL in all subjects, it failed to attain the 30 ng/mL threshold, even after three months of supplementation [26]. For this reason, higher doses would seem to be required. Indeed, the ones adopted in our trial were found to be effective in attaining the 30 ng/mL threshold, especially in the groups with a more refracted regimen. This specific aspect has significant clinical implications, as it could help in guiding the physician when adopting a higher threshold is advised [29].

After cholecalciferol discontinuation, 25 (OH)D levels remained over 30 ng/mL in all three groups for the remaining month of follow-up. Interestingly, after 112 days, the three regimens were found to be equally effective in maintaining a 25 (OH)D serum levels of at least 20 ng/mL, but only Groups A and B still completely maintained levels over 30 ng/mL. The comparison among AUCs corrected for the cumulative dose reached at the different observation time points confirmed a greater exposure associated with a more refracted schedule (higher for Group A, intermediate for Group B and lower for Group C), particularly evident when the follow-up after treatment discontinuation was included (day 84 to 112). This suggests an improved capacity of maintaining higher 25 (OH)D levels with a more frequent administration and may be of clinical relevance when the clinician considers it appropriate to achieve a reasonable buffer against insufficiency over time. In the future, it might be interesting to investigate the rate of decrease of 25 (OH)D serum levels over more extended time periods after supplementation discontinuation.

Our findings therefore provide further evidence for this intriguing hypothesis: The higher efficiency of the daily administration that we observed may indeed explain the improved clinical outcomes for daily vs. intermittent doses observed in some reports [9,15,16].

In terms of safety profile, cholecalciferol supplementation at the doses adopted in our trial proved to be excellent and no cases of hypercalcemia occurred. A statistically significant, though clinically negligible, increase in total calcium serum levels was found with respect to baseline only for Group B at day 56, day 84, and day 112. However, the consensual significant increase in serum albumin observed in Group B at day 56 might justify this observation. The possibility of a random fluctuation could indeed be a reasonable explanation. However, a direct effect of vitamin D on albumin synthesis cannot be ruled out. In the literature, there are data (observed in patients with severe kidney failure) suggesting that vitamin D deficiency might play a role in the impairment of albumin synthesis [30].

The safety of high-dose vitamin D supplementation such as those we used in this study (even after attainment of serum 25 (OH)D levels well above 30–40 ng/mL) could provide the rationale for clinical trials aimed to achieve these serum concentrations in order to assess whether they are associated with immunomodulatory effects of vitamin D in autoimmune diseases and/or with the prevention of infection risk [17,31,32].

Our study does have some limitations that need to be mentioned. Liquid chromatography-tandem mass spectrometry is currently considered to be the most accurate and precise method for measuring 25-hydroxyvitamin D, but we measured 25 (OH)D serum levels by immunoenzymatic reaction (ELISA), a method that can be affected by various interferences [33,34]. However, given the fact that we studied a healthy (although vitamin D deficient) population, we do not expect these issues to undermine the observed results. Nevertheless, for the same reason, one should use caution before generalizing our results to a diseased population (i.e., the frail elderly, patients affected by chronic illnesses such as chronic kidney disease, liver failure, malabsorption, etc.), because the absorption and/or the metabolism of vitamin D metabolites might be different in these settings. Finally, no genetic assessment was performed to investigate the possible interactions between the genetic profile and the response to vitamin D supplementation.

## 6. Conclusions

In summary, findings from the present study demonstrate that as well as maintaining an excellent safety profile, normalization of 25 (OH)D serum levels was quickly achieved with three different supplementation regimens. However, a more refracted schedule was observed to provide a higher systemic 25 (OH)D exposure. Further studies undertaken to investigate whether these observed differences in PK can explain the improved clinical outcomes seen with the daily administration compared to bolus-based regimens are warranted.

## Figures and Tables

**Table 1:** Baseline demographic and biochemical characteristics of subjects.

Clinical Characteristics *	All Subjects (*N* = 75)	Group A (*N* = 25)	Group B (*N* = 25)	Group C (*N* = 25)
Age (years)	34.1 ± 10.2	30.2 ± 10	36.7 ± 8.8	35.4 ± 11
Sex				
Male, *n* (%)	31 (41.3)	12 (48)	7 (28)	12 (48)
Female, *n* (%)	44 (58.7)	13 (52)	18 (72)	13 (52)
Weight (kg)	66.7 ± 12.4	65.2 ± 13.5	67.4 ± 9.8	67.6 ± 13.7)
BMI (kg/m^2^)	23.1 ± 2.6	22.6 ± 2.9	23.4 ± 2.1	23.2 ± 2.8)
25 (OH)D (ng/mL)	13.7 ± 3.8	14.7 ± 3.9	12.8 ±3	13.5 ± 4.1
Calcium (mmol/L)	2.3 ± 0.1	2.3 ± 0.1	2.3 ± 0.1	2.3 ± 0.1
Phosphate (mmol/L)	1.1 ± 0.1	1.1 ± 0.2	1.1 ± 0.1	1 ± 0.1
Albumin (g/L)	45 ± 2.6	45.3 ± 2.7	44.3 ± 2.8	45.4 ± 2.3

* No statistically significant difference was observed among groups. BMI = body mass index. Data presented as mean ± standard deviation or number (%).

**Table 2:** Pharmacokinetics parameters determined from the serum 25 (OH)D concentration.

Parameter	Group A	Group B	Group C	Ratio of Geometric Means Ratio (90% Confidence Interval)		
				A vs. B	A vs. C	B vs. C
C_max_	79.59	63.17	58.27	1.26 (1.16, 1.37)	1.37 (1.26, 1.49)	1.08 (1.0, 1.18)
AUC_0–84_	4648.09	3593.69	3374.12	1.29 (1.20, 1.39)	1.38 (1.28, 1.48)	1.07 (0.99, 1.15)
AUC_0–112_	5947.29	4982.70	4530.24	1.19 (1.11, 1.29)	1.31 (1.22, 1.41)	1.10 (1.02, 1.19)
AUC_84–112_	1295.45	1365.16	1188.11	0.95 (0.88, 1.03)	1.09 (1.01, 1.18)	1.15 (1.06, 1.25)

## Data Availability

Data sharing is not applicable to this article.

## Supplementary Materials

The following are available online at https://www.mdpi.com/2072-6643/12/6/1553/s1, Table S1: statistical analysis of pharmacokinetic parameters for 25(OH)D3 following repeated oral doses of cholecalciferol, Table S2: subjects with 25(OH)D levels ≥20 ng/mL and ≥30 ng/mL on Day 7, 14, 21, 28, 35, 42, 49, 56, 63, 77, 84, 85, 87, 89, 96, 103, 112, Table S3: pharmacodynamic parameters over time, Table S4: summary of adverse events during the study.
