# Supplementary material for: Pharmacokinetics of Oral Cholecalciferol in Healthy Subjects with Vitamin D Deficiency: A Randomized Open-Label Study
Source: Nutrients. 2020 May 27;12(6):1553. doi: 10.3390/nu12061553 (PMC7352201; doi:10.3390/nu12061553)
Supplement: Supplementary file 1 [file nutrients-12-01553-s001.pdf]

Supplementary Table S1. Statistical analysis of pharmacokinetic parameters for 25(OH)D3 following repeated oral doses of cholecalciferol**.**

Time	25(OH)D3 (ng/ml)	ALL subjects (n=73)	Group A (n=24)	Group B (n=25)	Group C (n=24)	ANOVA p-value
V2 - Baseline	N<br>Mean (SD)<br>Median (IQR)<br>Min-Max	73<br>13.5 (3.7)<br>13.8 (11.5-16)<br>5-21.9	24<br>14.4 (3.9)<br>14.7 (12.4-16.6)<br>7-21.9	25<br>12.8 (3)<br>12.6 (10.7-15.1)<br>5-18.4	24<br>13.4 (4.2)<br>13.4 (10-16.8)<br>5.9-19.8	0.319
V3 – Day 7	N<br>Mean (SD)<br>Median (IQR)<br>Min-Max	73<br>23.3 (4.4)<br>22.7 (20.2-26.2)<br>14.2-35.6	24<br>23.5 (3.6)<br>22.9 (20.6-26.5)<br>14.2-29.6	25<br>20.9 (3)<br>20.6 (19.2-22.8)<br>15.3-26.4	24<br>25.7 (5.2)<br>25.9 (22.2-28.1)<br>14.7-35.6	<0.001
V4 – Day 14	N<br>Mean (SD)<br>Median (IQR)<br>Min-Max	73<br>28.2 (5.4)<br>28.4 (25.1-31)<br>17.2-40.4	24<br>32.5 (5)<br>32.2 (29.8-36.6)<br>20.5-40.4	25<br>26.5 (3.4)<br>26.6 (25.1-29.3)<br>18.9-31.8	24<br>25.6 (4.9)<br>24.8 (22.5-28.7)<br>17.2-34.7	<0.001
V5 – Day 21	N<br>Mean (SD)<br>Median (IQR)<br>Min-Max	73<br>38 (9.2)<br>36.5 (31.7-42.4)<br>18.8-70	24<br>44.2 (10.9)<br>42.6 (36-51.6)<br>21-70	25<br>34.1 (5.3)<br>34.2 (30.7-37.5)<br>22.7-45.6	24<br>35.8 (7.6)<br>36.5 (30.4-40.1)<br>18.8-49.2	<0.001
V6 – Day 28	N<br>Mean (SD)<br>Median (IQR)<br>Min-Max	73<br>43.6 (11.1)<br>41.2 (36.1-48.8)<br>25.9-85.2	24<br>55.2 (10.2)<br>55.6 (47.6-62)<br>40.4-85.2	25<br>38.9 (4.2)<br>39.2 (36.4-41.2)<br>29.6-48.8	24<br>36.9 (7.2)<br>36 (32.1-41.3)<br>25.9-59.2	<0.001
V7 – Day 35	N<br>Mean (SD)<br>Median (IQR)<br>Min-Max	73<br>49.9 (12.9)<br>46.8 (40-56.8)<br>26.5-84	24<br>62.6 (13)<br>63.2 (54.7-71.8)<br>33.8-84	25<br>43.6 (5.5)<br>43.6 (39.4-47.2)<br>36.3-54.8	24<br>43.8 (8.3)<br>43.8 (39.4-47.8)<br>26.5-57.6	<0.001
V8 – Day 42	N<br>Mean (SD)<br>Median (IQR)<br>Min-Max	73<br>54.6 (15.2)<br>51.6 (44-62)<br>29.4-100.4	24<br>71 (13.4)<br>68.4 (62.5-80.1)<br>47.2-100.4	25<br>49.4 (5.4)<br>49.2 (46-52.4)<br>39-60	24<br>43.5 (8.5)<br>43 (36.6-49)<br>29.4-62	<0.001
V9 – Day 49	N<br>Mean (SD)<br>Median (IQR)<br>Min-Max	73<br>56.3 (15)<br>51.6 (46.8-65.2)<br>28-97.6	24<br>72.4 (12.9)<br>70.8 (61.9-79.8)<br>49.6-97.6	25<br>51.1 (6.2)<br>50 (47.2-53.2)<br>42-65.2	24<br>45.7 (9)<br>45.6 (40-51.6)<br>28-65.6	<0.001
V10 – Day 56	N<br>Mean (SD)<br>Median (IQR)<br>Min-Max	73<br>59.6 (17.9)<br>55.2 (47.2-68.4)<br>31-113.6	24<br>79.1 (16.2)<br>78.6 (67.8-92.3)<br>49.2-113.6	25<br>53.5 (7.2)<br>52.8 (48.4-60)<br>40.4-68.4	24<br>46.4 (8.2)<br>47 (42.9-52.3)<br>31-59.6	<0.001
V11 – Day 63	N<br>Mean (SD)<br>Median (IQR)<br>Min-Max	73<br>57.7 (13.3)<br>54.4 (49.2-66.8)<br>32.5-90.4	24<br>70.8 (11.5)<br>71.6 (61.7-79.6)<br>52-90.4	25<br>52.8 (7)<br>51.6 (49.2-56.4)<br>40.8-72.8	24<br>49.8 (10.1)<br>49.2 (45.1-55.6)<br>32.5-71.2	<0.001
V12 – Day 77	N<br>Mean (SD)<br>Median (IQR)<br>Min-Max	73<br>56.5 (10.8)<br>56 (48.8-63.2)<br>35.7-79.6	24<br>60.8 (11.6)<br>60.8 (53.3-70.2)<br>39.9-79.6	25<br>55.5 (7.8)<br>55.2 (48.8-60.8)<br>43.2-71.2	24<br>53.1 (11.6)<br>55 (43-60.2)<br>35.7-77.2	0.039
V13 – Day 84	N<br>Mean (SD)<br>Median (IQR)<br>Min-Max	72<br>55.1 (10.2)<br>53.8 (48.7-62.4)<br>33.2-80	24<br>56.9 (10.7)<br>54.8 (51.8-62.1)<br>40-80	24<br>58.2 (9.2)<br>57.2 (50.8-66)<br>43.6-74.8	24<br>50.3 (9.3)<br>49.6 (43.3-57.4)<br>33.2-66.4	0.014
V14 – Day 85	N<br>Mean (SD)	67<br>55.3 (10.5)	24<br>57.3 (11.5)	21<br>58.6 (8.2)	22<br>49.9 (9.6)	0.012

	Median (IQR)<br>Min-Max	56.8 (48.6-61.4)<br>32.2-77.6	57.2 (52.1-62.6)<br>32.2-77.6	60 (53.2-64.8)<br>42.4-76.8	49.2 (44.7-58.9)<br>33.7-64	
V15 – Day 87	N<br>Mean (SD)<br>Median (IQR)<br>Min-Max	69<br>52.1 (10.1)<br>53.2 (46-58)<br>27.4-78.8	24<br>53.6 (11.5)<br>52.6 (47.6-57.8)<br>27.4-78.8	23<br>55.4 (6.6)<br>56 (52.8-59.4)<br>42.4-68.4	22<br>47 (10)<br>46.2 (40.9-53.2)<br>29-70	0.012
V16 - Day 89	N<br>Mean (SD)<br>Median (IQR)<br>Min-Max	69<br>49.7 (9.6)<br>49.6 (41.6-56.4)<br>30.6-69.6	24<br>49.9 (8.7)<br>50.6 (43.4-55.4)<br>34.7-68.4	23<br>54.1 (9.5)<br>53.2 (49.6-60)<br>30.6-69.6	22<br>45 (8.9)<br>45.2 (37.4-51)<br>33.8-63.2	0.006
V17 - Day 96	N<br>Mean (SD)<br>Median (IQR)<br>Min-Max	69<br>49.7 (11.2)<br>48.8 (41.2-55.2)<br>30.8-89.5	24<br>49.2 (9.7)<br>50.8 (41.2-55.6)<br>30.8-70	23<br>53 (10)<br>50.8 (47.6-55.2)<br>37.4-76.8	22<br>46.9 (13.2)<br>44.2 (39.4-49.4)<br>32.2-89.5	0.184
V18* Day 103	N<br>Mean (SD)<br>Median (IQR)<br>Min-Max	69<br>41.1 (9.4)<br>40.4 (34.3-47.2)<br>25.6-68	24<br>41.1 (8.1)<br>41 (35.3-46.3)<br>25.6-58.8	23<br>44.3 (10.5)<br>40.8 (36.2-51.6)<br>27-68	22<br>37.8 (8.8)<br>35.9 (30.7-40.8)<br>25.6-60.4	0.065
V19* - Day 112	N<br>Mean (SD)<br>Median (IQR)<br>Min-Max	69<br>43.5 (8.1)<br>42.4 (37.5-49.6)<br>27.6-64.4	24<br>44.4 (9.1)<br>42.6 (37.5-50.3)<br>31.6-64.4	23<br>45.3 (7.5)<br>44.4 (38.8-49.6)<br>34.4-61.6	22<br>40.8 (7.1)<br>39.4 (36-43.3)<br>27.6-56	0.15

Supplementary Table S2. Subjects with 25(OH)D levels ≥ 20 ng/mL and ≥ 30 ng/mL on Day 7, 14, 21, 28, 35, 42, 49, 56, 63, 77, 84, 85, 87, 89, 96, 103, 112.

Levels	ALL subjects (n=71)	Group A (n=24)	Group B (n=23)	Group C (n=24)
**≥20ng/mL**				
Number of subjects with 25-OH-D <20ng/ml at baseline	69	22	23	24
Number of subjects with 25-OH-D ≥20ng/ml on Day 7	55/69 (79.7%)	19/22 (86.3%)	15/23 (65.2%)	21/24 (87.5%)
Number of subjects with 25-OH-D ≥20ng/ml on Day 14	65/69 (94.2%)	22/22 (100%)	22/23 (95.6%)	21/24 (87.5%)
Number of subjects with 25-OH-D ≥20ng/ml on Day 21	68/69 (98.6%)	22/22(100%)	23/23 (100%)	23/24 (95.8%)
Number of subjects with 25-OH-D ≥20ng/ml on Day 28	69/69 (100%)	22/22 (100%)	23/23 (100%)	24/24 (100%)
Number of subjects with 25-OH-D ≥20ng/ml on Day 35	69/69 (100%)	22/22 (100%)	23/23 (100%)	24/24 (100%)
Number of subjects with 25-OH-D ≥20ng/ml on Day 42	69/69 (100%)	22/22 (100%)	23/23 (100%)	24/24 (100%)
Number of subjects with 25-OH-D ≥20ng/ml on Day 49	69/69 (100%)	22/22 (100%)	23/23 (100%)	24/24 (100%)
Number of subjects with 25-OH-D ≥20ng/ml on Day 56	69/69 (100%)	22/22 (100%)	23/23 (100%)	24/24 (100%)
Number of subjects with 25-OH-D ≥20ng/ml on Day 63	69/69 (100%)	22/22 (100%)	23/23 (100%)	24/24 (100%)
Number of subjects with 25-OH-D ≥20ng/ml on Day 77	69/69 (100%)	22/22 (100%)	23/23 (100%)	24/24 (100%)
Number of subjects with 25-OH-D ≥20ng/ml on Day 84	*69/69 (100%)*	*22/22 (100%)*	*23/23 (100%)*	*24/24 (100%)*
Number of subjects with 25-OH-D ≥20ng/ml on Day 85*	65/65 (100%)*	22/22 (100%)*	21/21 (100%)*	22/22 (100%)*
Number of subjects with 25-OH-D ≥20ng/ml on Day 87*	65/65 (100%)*	22/22 (100%)*	21/21 (100%)*	22/22 (100%)*
Number of subjects with 25-OH-D ≥20ng/ml on Day 89*	65/65 (100%)*	22/22 (100%)*	21/21 (100%)*	22/22 (100%)*
Number of subjects with 25-OH-D ≥20ng/ml on Day 96*	65/65 (100%)*	22/22 (100%)*	21/21 (100%)*	22/22 (100%)*
Number of subjects with 25-OH-D ≥20ng/ml on Day 103*	65/65 (100%)*	22/22 (100%)*	21/21 (100%)*	22/22 (100%)*
Number of subjects with 25-OH-D ≥20ng/ml on Day 112*	65/65 (100%)*	22/22 (100%)*	21/21 (100%)*	22/22 (100%)*
**≥30ng/mL**				
Number of subjects with 25-OH-D <30ng/ml at baseline	71	24	23	24
Number of subjects with 25-OH-D ≥30ng/ml on Day 7	3/71 (4.2%)	0/24 (0%)	0/23 (0%)	3/24 (12.5%)
Number of subjects with 25-OH-D ≥30ng/ml on Day 14	26/71 (36.6%)	17/24 (70.8%)	4/23 (17.4%)	5/24 (20.8%)
Number of subjects with 25-OH-D ≥30ng/ml on Day 21	59/71 (83.1%)	22/24 (91.7%)	18/23 (78.3%)	19/24 (79.2%)
Number of subjects with 25-OH-D ≥30ng/ml on Day 28	66/71 (93%)	24/24 (100%)	22/23 (95.7%)	20/24 (83.3%)
Number of subjects with 25-OH-D ≥30ng/ml on Day 35	70/71 (98.6%)	24/24 (100%)	23/23 (100%)	23/24 (95.8%)
Number of subjects with 25-OH-D ≥30ng/ml on Day 42	70/71 (98.6%)	24/24 (100%)	23/23 (100%)	23/24 (95.8%)
Number of subjects with 25-OH-D ≥30ng/ml on Day 49	70/71 (98.6%)	24/24 (100%)	23/23 (100%)	23/24 (95.8%)
Number of subjects with 25-OH-D ≥30ng/ml on Day 56	71/71 (100%)	24/24 (100%)	23/23 (100%)	24/24 (100%)
Number of subjects with 25-OH-D ≥30ng/ml on Day 63	71/71 (100%)	24/24 (100%)	23/23 (100%)	24/24 (100%)
Number of subjects with 25-OH-D ≥30ng/ml on Day 77	71/71 (100%)	24/24 (100%)	23/23 (100%)	24/24 (100%)
Number of subjects with 25-OH-D ≥30ng/ml on Day 84	*71/71 (100%)*	*24/24 (100%)*	*23/23 (100%)*	*24/24 (100%)*
Number of subjects with 25-OH-D ≥30ng/ml on Day 85**	67/67 (100%)**	24/24 (100%)**	21/21 (100%)**	22/22 (100%)**
Number of subjects with 25-OH-D ≥30ng/ml on Day 87**	65/67 (97%)**	23/24 (95.8%)**	21/21 (100%)**	21/22 (95.5%)**
Number of subjects with 25-OH-D ≥30ng/ml on Day 89**	67/67 (100%)**	24/24 (100%)**	21/21 (100%)**	22/22 (100%)**
Number of subjects with 25-OH-D ≥30ng/ml on Day 96**	67/67 (100%)**	24/24 (100%)**	21/21 (100%)**	22/22 (100%)**
Number of subjects with 25-OH-D ≥30ng/ml on Day 103**	63/67 (94%)**	23/24 (95.8%)**	20/21 (95.2%)**	20/22 (90.9%)**
Number of subjects with 25-OH-D ≥30ng/ml on Day 112**	66/67 (98.5%)**	24/24 (100%)**	21/21 (100%)**	21/22 (95.5%)**

* 4 subjects who took a dose on Day 84 (not per-protocol) are excluded from statistics of V14-V19. Percentages computed on 65 (69-4)

** 4 subjects who took a dose on Day 84 (not per-protocol) are excluded from statistics of V14-V19. Percentages computed on 67 (71-4)

Supplementary Table S3. Pharmacodynamic parameters over time

Time		ALL subjects (n=73)	Group A (n=24)	Group B (n=25)	Group C (n=24)	ANOVA p-value
**Calcium**						
V2	N<br>Mean (SD)<br>Median (IQR)<br>Min-Max	73<br>2.3 (0.1)<br>2.3 (2.2-2.4)<br>2.2-2.5	24<br>2.3 (0.1)<br>2.3 (2.3-2.4)<br>2.2-2.5	25<br>2.3 (0.1)<br>2.3 (2.2-2.3)<br>2.2-2.4	24<br>2.3 (0.1)<br>2.3 (2.2-2.4)<br>2.2-2.5	0.372
V6	N<br>Mean (SD)<br>Median (IQR)<br>Min-Max	73<br>2.3 (0.1)<br>2.3 (2.3-2.4)<br>2.1-2.5	24<br>2.3 (0.1)<br>2.3 (2.3-2.3)<br>2.1-2.5	25<br>2.3 (0.1)<br>2.3 (2.3-2.4)<br>2.2-2.4	24<br>2.3 (0.1)<br>2.3 (2.3-2.4)<br>2.2-2.5	0.285
V10	N<br>Mean (SD)<br>Median (IQR)<br>Min-Max	73<br>2.3 (0.1)<br>2.3 (2.3-2.4)<br>2.2-2.5	24<br>2.3 (0.1)<br>2.4 (2.3-2.4)<br>2.2-2.5	25<br>2.3 (0.1)<br>2.3 (2.3-2.4)<br>2.2-2.5	24<br>2.3 (0.1)<br>2.3 (2.3-2.4)<br>2.2-2.5	0.956
V13	N<br>Mean (SD)<br>Median (IQR)<br>Min-Max	72<br>2.3 (0.1)<br>2.3 (2.3-2.4)<br>2.1-2.5	24<br>2.3 (0.1)<br>2.3 (2.3-2.4)<br>2.2-2.4	24<br>2.3 (0.1)<br>2.3 (2.3-2.4)<br>2.1-2.5	24<br>2.3 (0.1)<br>2.3 (2.3-2.4)<br>2.2-2.4	0.958
V19*	N<br>Mean (SD)<br>Median (IQR)<br>Min-Max	68<br>2.3 (0.1)<br>2.3 (2.3-2.4)<br>2.2-2.5	23<br>2.3 (0.1)<br>2.3 (2.3-2.3)<br>2.2-2.5	23<br>2.3 (0.1)<br>2.3 (2.2-2.3)<br>2.2-2.5	22<br>2.3 (0.1)<br>2.4 (2.3-2.4)<br>2.2-2.5	0.396
**Ionized calcium**						
V2	N<br>Mean (SD)<br>Median (IQR)<br>Min-Max	73<br>1.2 (0)<br>1.2 (1.2-1.2)<br>1.2-1.3	24<br>1.2 (0)<br>1.2 (1.2-1.3)<br>1.2-1.3	25<br>1.2 (0)<br>1.2 (1.2-1.2)<br>1.2-1.3	24<br>1.2 (0)<br>1.2 (1.2-1.2)<br>1.2-1.3	0.208
V6	N<br>Mean (SD)<br>Median (IQR)<br>Min-Max	72<br>1.2 (0)<br>1.2 (1.2-1.2)<br>1-1.3	24<br>1.2 (0)<br>1.2 (1.2-1.2)<br>1-1.3	24<br>1.2 (0)<br>1.2 (1.2-1.2)<br>1.2-1.3	24<br>1.2 (0)<br>1.2 (1.2-1.2)<br>1.2-1.3	0.636
V10	N<br>Mean (SD)<br>Median (IQR)<br>Min-Max	73<br>1.2 (0)<br>1.2 (1.2-1.3)<br>1.2-1.3	24<br>1.2 (0)<br>1.2 (1.2-1.3)<br>1.2-1.3	25<br>1.2 (0)<br>1.2 (1.2-1.3)<br>1.2-1.3	24<br>1.2 (0)<br>1.2 (1.2-1.3)<br>1.2-1.3	0.486
V13	N<br>Mean (SD)<br>Median (IQR)<br>Min-Max	72<br>1.2 (0)<br>1.2 (1.2-1.2)<br>1.2-1.3	24<br>1.2 (0)<br>1.2 (1.2-1.2)<br>1.2-1.3	24<br>1.2 (0)<br>1.2 (1.2-1.2)<br>1.2-1.3	24<br>1.2 (0)<br>1.2 (1.2-1.2)<br>1.2-1.3	0.674
V19*	N<br>Mean (SD)<br>Median (IQR)<br>Min-Max	69<br>1.2 (0)<br>1.2 (1.2-1.2)<br>1.1-1.3	24<br>1.2 (0)<br>1.2 (1.2-1.2)<br>1.2-1.3	23<br>1.2 (0)<br>1.2 (1.2-1.2)<br>1.2-1.3	22<br>1.2 (0)<br>1.2 (1.2-1.2)<br>1.1-1.3	0.373
**Albumin**						
V2	N<br>Mean (SD)<br>Median (IQR)<br>Min-Max	73<br>44.9 (2.6)<br>45.3 (43-46.8)<br>40.1-52.6	24<br>45.2 (2.8)<br>45.4 (43.3-47.4)<br>40.3-49.6	25<br>44.3 (2.8)<br>44.2 (42.2-45.8)<br>40.1-52.6	24<br>45.3 (2.2)<br>45.1 (43.4-47.3)<br>42.1-48.9	0.323
V6	N<br>Mean (SD)<br>Median (IQR)<br>Min-Max	73<br>45.1 (3)<br>45.1 (43-46.6)<br>36.7-52.1	24<br>45.4 (4)<br>45.7 (42.1-48.4)<br>36.7-52.1	25<br>44.5 (2.6)<br>44.3 (42.7-46.1)<br>38.7-52	24<br>45.5 (2.1)<br>45.3 (44.4-46.8)<br>42.2-50.1	0.427
V10	N<br>Mean (SD)<br>Median (IQR)<br>Min-Max	73<br>45.3 (2.7)<br>45.1 (43.4-46.6)<br>40.5-53.7	24<br>46 (2.4)<br>46 (43.7-48)<br>41.7-49.9	25<br>45.1 (2.9)<br>44.7 (42.9-46.3)<br>40.6-53.7	24<br>44.9 (2.7)<br>44.7 (43.1-45.3)<br>40.5-51.9	0.354
V13	N<br>Mean (SD)<br>Median (IQR)<br>Min-Max	72<br>44.7 (3.1)<br>44.8 (42.6-47.2)<br>34.5-52.1	24<br>45 (3.1)<br>45 (42.6-47.5)<br>38.8-50.2	24<br>44.4 (3.6)<br>44.9 (42.5-46)<br>34.5-52.1	24<br>44.8 (2.5)<br>44 (42.6-47.1)<br>41.2-48.7	0.813

V19*	N<br>Mean (SD)<br>Median (IQR)<br>Min-Max	69<br>44.4 (2.6)<br>44.7 (42.7-45.5)<br>39-52.6	24<br>44.3 (2.3)<br>44.9 (43.2-45.5)<br>39.2-48.7	23<br>44.3 (3.4)<br>43.7 (42.1-45.2)<br>39-52.6	22<br>44.6 (1.9)<br>44.7 (43.1-45.5)<br>41.6-49.7	0.909
**Phosphate**						
V2	N<br>Mean (SD)<br>Median (IQR)<br>Min-Max	73<br>1.1 (0.2)<br>1.1 (1-1.1)<br>0.6-1.6	24<br>1.1 (0.2)<br>1.1 (1-1.2)<br>0.7-1.5	25<br>1.1 (0.1)<br>1.1 (1-1.1)<br>0.9-1.2	24<br>1.1 (0.2)<br>1.1 (1-1.1)<br>0.6-1.6	0.85
V6	N<br>Mean (SD)<br>Median (IQR)<br>Min-Max	73<br>1.1 (0.1)<br>1.1 (1-1.2)<br>0.7-1.5	24<br>1.1 (0.1)<br>1.1 (1-1.2)<br>0.9-1.4	25<br>1.1 (0.1)<br>1.1 (1-1.2)<br>0.8-1.3	24<br>1.1 (0.2)<br>1.1 (1-1.2)<br>0.7-1.5	0.503
V10	N<br>Mean (SD)<br>Median (IQR)<br>Min-Max	73<br>1.1 (0.2)<br>1.1 (1-1.2)<br>0.7-1.5	24<br>1.1 (0.2)<br>1.1 (1-1.2)<br>0.8-1.5	25<br>1.1 (0.1)<br>1.1 (1-1.2)<br>0.8-1.4	24<br>1.1 (0.2)<br>1.1 (1-1.2)<br>0.7-1.5	0.539
V13	N<br>Mean (SD)<br>Median (IQR)<br>Min-Max	72<br>1.1 (0.1)<br>1.1 (1-1.2)<br>0.7-1.4	24<br>1.1 (0.1)<br>1.1 (1-1.2)<br>0.7-1.4	24<br>1.1 (0.1)<br>1.1 (1-1.1)<br>0.8-1.3	24<br>1.1 (0.2)<br>1.1 (1.1-1.2)<br>0.7-1.4	0.267
V19*	N<br>Mean (SD)<br>Median (IQR)<br>Min-Max	69<br>1.1 (0.1)<br>1.1 (1-1.1)<br>0.7-1.4	24<br>1 (0.1)<br>1 (1-1.1)<br>0.9-1.3	23<br>1.1 (0.1)<br>1 (1-1.1)<br>0.8-1.4	22<br>1.1 (0.2)<br>1.1 (1-1.1)<br>0.7-1.4	0.839

Supplementary Table S4. Summary of adverse events during the study

	All AE (N)	All Subject N (%)	Group A AE (N)	Group A Subject N (%)	Group B AE (N)	Group B Subject N(%)	Group C AE (N)	Group C Subject N (%)
AEs	118	49 (65.3)	42	18 (72)	48	19 (76)	28	12 (48)
Mild AEs	86	38 (50.7)	33	15 (60)	34	13 (52)	19	10 (40)
Moderate AEs	32	25 (33.3)	9	8 (32)	14	10 (40)	9	7 (28)
Severe AEs	0	0 (0)	0	0 (0)	0	0 (0)	0	0 (0)
SAEs	0	0 (0)	0	0 (0)	0	0 (0)	0	0 (0)
IMP-related AEs (certain/probable/possible)	1	1 (1.3)	1	1 (4)	0	0 (0)	0	0 (0)
IMP-not related AEs (no reasonable causal relationship)*	117	49 (65.3)	41	18 (72)	48	19 (76)	28	12 (48)
AEs leading to withdrawal	1	1 (1.3)	1	1 (4)	0	0 (0)	0	0 (0)

IMP: medicinal product (cholecalciferol)
AE n: number of adverse events
Subject n: number of subjects with AE
** considering 'not related' and 'unlikely relationship'*
